# The PI3K/mTOR inhibitor Gedatolisib eliminates dormant breast cancer cells in organotypic culture, but fails to prevent metastasis in preclinical settings

**DOI:** 10.1002/1878-0261.13031

**Published:** 2021-06-25

**Authors:** Ryann E. Shor, Jinxiang Dai, Sun‐Young Lee, Laura Pisarsky, Irina Matei, Serena Lucotti, David Lyden, Mina J. Bissell, Cyrus M. Ghajar

**Affiliations:** ^1^ Public Health Sciences Division/Translational Research Program Fred Hutchinson Cancer Research Center Seattle WA USA; ^2^ Biological Systems and Engineering Division Lawrence Berkeley National Laboratory CA USA; ^3^ Children’s Cancer and Blood Foundation Laboratories Department of Pediatrics, and Cell and Developmental Biology Drukier Institute for Children’s Health Meyer Cancer Center Weill Cornell Medicine New York NY USA; ^4^ Human Biology Division Fred Hutchinson Cancer Research Center Seattle WA USA

**Keywords:** breast cancer, disseminated tumor cell dormancy, gedatolisib, integrin‐β1, metastasis, phosphatidylinositol 3‐kinase/PI3K

## Abstract

Dormant, disseminated tumor cells (DTCs) are thought to be the source of breast cancer metastases several years or even decades after initial treatment. To date, a selective therapy that leads to their elimination has not been discovered. While dormant DTCs resist chemotherapy, evidence suggests that this resistance is driven not by their lack of proliferation, but by their engagement of the surrounding microenvironment, via integrin‐β1‐mediated interactions. Because integrin‐β1‐targeted agents have not been translated readily to the clinic, signaling nodes downstream of integrin‐β1 could serve as attractive therapeutic targets in order to sensitize dormant DTCs to therapy. By probing a number of kinases downstream of integrin‐β1, we determined that PI3K inhibition with either a tool compounds or a compound (PF‐05212384; aka Gedatolisib) in clinical trials robustly sensitizes quiescent breast tumor cells seeded in organotypic bone marrow cultures to chemotherapy. These results motivated the preclinical study of whether Gedatolisib—with or without genotoxic therapy—would reduce DTC burden and prevent metastases. Despite promising results in organotypic culture, Gedatolisib failed to reduce DTC burden or delay, reduce or prevent metastasis in murine models of either triple‐negative or estrogen receptor‐positive breast cancer dissemination and metastasis. This result held true whether analyzing Gedatolisib on its own (vs. vehicle‐treated animals) or in combination with dose‐dense doxorubicin and cyclophosphamide (vs. animals treated only with dose‐dense chemotherapies). These data suggest that PI3K is not the node downstream of integrin‐β1 that confers chemotherapeutic resistance to DTCs. More broadly, they cast doubt on the strategy to target PI3K in order to eliminate DTCs and prevent breast cancer metastasis.

## Graphical abstract

Dormant disseminated tumor cells (DTCs) are a potential source of late distant breast cancer recurrences. Previously, we identified integrin‐β1 as a promising target for DTC chemosensitization and metastasis prevention. Here, we explore downstream kinases, and find that despite promising initial results, targeting PI3K lacks efficacy on its own or in combination with chemotherapy in two different pre‐clinical models.

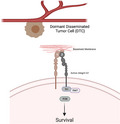

**Abbreviations**
AC: adriamycin and cyclophosphamide
BLI: bioluminescent imaging
bm‐MVNs: bone marrow microvascular niches
DAPI: 4′,6‐Diamidine‐2′‐phenylindole dihydrochloride
DTCs: disseminated tumor cells
ECs: human umbilical vein endothelial cells
ER^+^
: estrogen receptor‐positive
ERK: ras‐extracellular signal‐regulated kinases
FAK: focal adhesion kinase
ffluc: firefly luciferase
ic: intracardiac
LrECM: laminin‐rich extracellular matrix
MFS: metastasis‐free survival
MSCs: bone marrow‐derived human mesenchymal stem cells
PFA: paraformaldehyde
PI3K: phosphatidylinositol 3‐kinase
PVN: perivascular niche
SFKs: src family kinases
T4‐2: HMT‐3522‐T4‐2
TNBC: triple‐negative breast cancer
YFP: yellow fluorescent protein

## 1 Introduction

Although advances in chemotherapies, endocrine therapies, and targeted therapies have dramatically improved the 5‐year survival rate of breast cancer, as many as 24% of patients will die of recurrent breast cancer 6 years or more following treatment, with ~ 10% of recurrences taking place a decade after initial treatment [1]. More recent meta‐analysis of women treated with endocrine therapy for 5 years demonstrated that the majority of recurrences took place 5 years after the onset of treatment [2]. Combined with a static rate of change in breast cancer mortality, these data suggest current therapies are delaying metastases, not halting them altogether.

Metastatic recurrence has been attributed to disseminated tumor cells (DTCs) that leave the primary site and extravasate into distant organs (e.g., bone marrow, lung, or liver), where they may die, steadily proliferate and emerge as overt metastases, or persist in a quiescent state. DTCs have been detected in the bone marrow of at least one‐quarter of early‐stage breast cancer patients at the time of diagnosis [3, 4]. The detection of DTCs in bone marrow aspirates predicts reduced metastasis‐free and overall survival, whereas their elimination is associated with enhanced metastasis‐free survival (MFS) in preclinical models and in humans [5, 6, 7]. Therefore, an approach to selectively eliminate DTCs could result in metastasis prevention. Still, we lack a defined clinical means to accomplish this.

We and others have shown previously that disseminated breast tumor cells reside within the bone marrow’s perivascular niche (PVN) [5, 7], where ‘angiocrine [8, 9]’ factors drive them into quiescence while promoting their long‐term survival [10, 11, 12, 13, 14]. Quiescent DTCs persist following chemotherapy [3, 5, 6, 15]. It was traditionally thought that DTCs in this state are resistant to chemotherapy due to cell cycle arrest [16]. We demonstrated recently that sensitivity to chemotherapy is not determined by cell cycle status, showing instead that the DTC microenvironment is responsible for chemoprotection [5]. We identified vascular cell adhesion molecule (VCAM)‐1 as a niche‐derived, chemoprotective factor and demonstrated that targeting integrin‐β1‐VCAM‐1 interactions sensitizes DTCs to chemotherapy, substantially decreases DTC burden *in* *vivo*, and prevents bone metastases in an estrogen receptor‐positive (ER^+^) model of breast cancer metastasis [5]. This study identified function blocking antibodies targeting integrin‐β1as a promising potential therapy for targeting dormant DTCs. But since there are no clinically approved integrin‐β1‐targeted therapies available for patients, this approach will require significant time and effort for clinical translation.

Integrin‐β1 is one subunit of a heterodimer that transduces physical and biochemical cues from extracellular ligand to regulate diverse cellular behaviors spanning proliferation, migration, and survival [17, 18]. Ligand binding causes integrin clustering and the activation of downstream signaling cascades encompassing focal adhesion kinase (FAK), Src family kinases (SFKs), Ras‐extracellular signal‐regulated kinases (ERK), and phosphatidylinositol 3‐kinase (PI3K)/AKT [17, 18]. A number of studies have directly implicated integrins or downstream mediators in chemotherapeutic resistance [17, 19, 20, 21, 22, 23, 24, 25, 26, 27], including in the context of tumor dormancy [5]. Because many putative effectors of integrin‐mediated chemoresistance have clinically approved compounds in the pipeline, it was of interest to identify more readily targetable signaling axes to trial in the context of minimal residual disease.

In this article, we examine downstream integrin signaling hubs with the aim of identifying a molecule that can be targeted with clinically approved compounds. Using small molecule inhibitors, we show that PI3K and Src are putative effectors of pro‐survival integrin signaling in organotypic culture. Using more clinically relevant compounds, we show that PI3K/mTOR inhibition by Gedatolisib results in DTC chemosensitization in organotypic culture. However, despite these promising results, Gedatolisib does not reduce DTC burden or improve metastasis‐free survival in mouse models of ER^+^ or triple‐negative breast cancer (TNBC). These data suggest that PI3K is not a key effector of pro‐survival signaling in DTCs *in* *vivo* and that other hubs downstream of integrin‐β1 are more critical to conferring cues that promote survival over the course of genotoxic therapy. More broadly, these results cast doubt on the strategy of employing PI3K inhibition (PI3Ki) in the context of minimal residual disease.

## 2 Methods

### 2.1 Cell culture and reagents

Freshly isolated human umbilical vein endothelial cells (ECs) were provided kindly by Dr. Andrew Putnam (University of Michigan) and propagated in fully supplemented EGM‐2 growth medium (Lonza). ECs were processed from umbilical cords obtained by a process considered exempt by the University of Michigan’s institutional review board (notice of determination dated August 21, 2014) because the tissue is normally discarded, and no identifying information is provided to the researchers who receive the cords. Bone marrow‐derived human mesenchymal stem cells (MSCs) were obtained commercially (ScienCell) and propagated in low glucose Dulbecco’s modified Eagle’s medium (DMEM) supplemented with 10% fetal bovine serum (Thermo Fisher, Waltham, CA, USA). All primary human cells were used in experiments by passage 11. Malignant HMT‐3522‐T4–2 (T4–2) cells were grown in H14 medium on collagen‐coated tissue culture flasks [28]. MCF‐7 cells were grown in high glucose DMEM supplemented with 10% FBS.

mCherry‐E4orf1‐ECs were generated as described previously [12, 29]. Yellow fluorescent protein (YFP) expressing T4–2 and MCF‐7 was generated by infection of tumor cells with pLentiCMV/YFP lentivirus followed by selection for 96 h with 1 μg·mL^−1^ puromycin.

#### 2.1.1 Generation of bone marrow microvascular niches

Bone marrow microvascular niches (bm‐MVNs) were generated as previously detailed [12]. MSCs were seeded at a density of 5 × 10^4^ cells·well^−1^ in 96‐well culture plates with mCherry‐E4‐ECs at a 5 : 1 ratio to generate bm‐MVNs. Cells were suspended in EGM‐2 at a concentration 6 × 10^4^ cells·100 μL^−1^. After depositing 100 μL of cellular suspension per well of a 96‐well plate, plates were left undisturbed on a flat surface for 15 min to allow even cell seeding prior to incubation. EGM‐2 media were then changed every 72 h.

After 10 days, YFP tumor cells were suspended in unsupplemented DMEM/F12 (300–800 cells·mL^−1^). Prior to counting, MCF‐7 cells were passaged three times through a 27‐gauge needle to obtain a single‐cell suspension. YFP tumor cells were seeded (100 μL·well^−1^) after washing cultures thrice with PBS. Cells were allowed to settle for 15 min at room temperature; then, a drip of laminin‐rich extracellular matrix (LrECM; Trevigen Cultrex Original basement membrane extract) in DMEM/F12 was slowly added to each well (final concentration: 20%). The LrECM drip condensed for 15 min at room temperature before polymerizing fully at 37 °C. Cultures were imaged at day 12 and day 17 post‐tumor‐cell seeding on a Zeiss LSM700 confocal microscope using a 0.3 NA 10 × air objective. The objective was centered to each well before acquisition of 512 × 512 pixel, 6 × 6 tiles (zoom = 0.7) that captured the near‐entirety of each well. Cultures were maintained with media changes every 72 h.

#### 2.1.2 Treatment with small molecule inhibitors and Gedatolisib

Small molecule signaling inhibitors were dissolved in DMSO to generate stock solutions with starting concentrations as follows: MAPKi 10 mm, PI3Ki 8 mm, SRCi 10 mm, JNKi 20 mm, PAKi 1 mm, FAKi 1 mm, IKKAα + βi 10 mm, IKKβ 10 mm, IKKɛ 1 mm. The appropriate volume of stock solution was added to 1 mL of DMEM/F12 to generate working concentrations as follows: MAPKi 10 μm, PI3Ki 8 μm, SRCi 10 μm, JNKi 10 μm, PAKi 1 μm, FAKi 1 μm, IKKα + βi 10 μm, IKKβi 10 μm, IKKɛi 1 μm. Doxorubicin (Tocris BioScience, Minneapolis, MN, USA) was diluted in ddH20 to generate 2500 mm stocks, which were further diluted 1 : 1000 in DMEM/F12 prior to treatment. Cultures were treated with 100 μL of inhibitor on Day ‐10 and 50 μL of (2×) inhibitor followed 1 h later with 50 μL doxorubicin on Day ‐12 and Day ‐15 postseeding of tumor cells. DMSO was used at 1 : 1000 as a vehicle control. Inhibitors, along with their sources and catalogue numbers, are listed below:

**Table:** 

Inhibitor	Drug name	Manufacturer	Catalogue number
MAPKi	PD98059	Cell Signaling Technologies (Danvers, MA, USA)	9900s
Pi3Ki	LY294002	Stem Cell Technologies	72 152
SRCi	PP2	Calbiochem	529 576
JNKi	SP200125	InvivoGen	tlrl‐sp60
PAKi	IPA‐3	Cayman	14 759
FAKi	Defactinib (VS‐6063)	Selleckchem	S7654
IKK A + Bi	IKK16	Selleckchem	S2882
IKKBi	BI605906	Tocris	5300
IKKEi	GSK 319347A	Tocris	5672

Gedatolisib (Selleckchem, S2628) was dissolved in DMSO to generate 1 mm stocks. Bm‐MVNs were primed with a dose curve of 6 doses of Gedatolisib ranging from 0.004 to 1 μm. Note that the LrECM drip further dilutes drug concentrations 1 : 2. Gedatolisib treatment was repeated on Day 12 and Day 15 postseeding. Bm‐MVNs were imaged on Day 12 prior to treatment and again on Day 17. If applicable, bm‐MVN cultures received 2× Gedatolisib treatment followed by 2× doxorubicin solution treatment exactly 1 h later on Day 12 and Day 15 postseeding. MVNs were fixed on Day 17 as described below.

#### 2.1.3 Immunofluorescent/TUNEL staining of microvascular niches

bm‐MVNs were fixed in 4% paraformaldehyde (PFA)/PBS for 15 min (4 °C), washed three times with ice cold PBS, and left in 0.5 m Glycine/PBS until staining (4 °C) in order to neutralize any unreacted PFA.

For staining, cultures were washed once with PBS prior to permeabilization in 0.5% Triton X‐100 (Sigma, X100)/PBS for 15 min (RT) and then washed three more times with PBS. TUNEL staining was conducted using a two‐component *In* *Situ* Cell Death Detection Kit (Roche/Sigma, 12156792910, St. Louis, MO, USA) that was thawed and mixed on ice according to manufacturer’s instructions. 30 μL of staining solution was added to each well prior to incubation for 1 h (37 °C). Cultures were washed three times in PBS prior to proceeding with immunofluorescent staining.

Cultures were blocked in 5% BSA/PBS (0.2 μm filtered) for 1 h (RT) prior to staining in primary antibody diluted in blocking solution overnight with gentle tilt‐shaking (4 °C). A rabbit anti‐pan‐cytokeratin (Abcam, ab9377, 1 : 500, Cambridge, MA, USA) primary antibody was used to visualize breast tumor cells. Cultures were washed the following morning in PBS (six washes over 1 h) prior to staining with a goat anti‐rabbit 488 (Invitrogen, A11008, 1 : 500, Waltham, MA, USA) secondary antibody diluted in blocking solution for 4–6 h (RT). 4′,6‐Diamidine‐2′‐phenylindole dihydrochloride (DAPI) was added to this solution (2 μg·mL^−1^) to visualize nuclei. Finally, cultures were washed extensively with PBS, culminating in an overnight wash prior to storage in PBS + 0.02% sodium azide.

### 2.2 Quantification of tumor cell growth, proliferative status, and apoptosis in microvascular niches

#### 2.2.1 Quantification of normalized tumor cell area fraction

A macro was written using NIH imagej software to remove bias from data quantification. For the YFP channel only day 12 and 17 images (i.e., prior to chemotherapeutic treatment, and post‐treatment) a constant threshold was applied to all samples within a given experiment to eliminate variability. The total area fraction of the 6 × 6 tiled image occupied by YFP^+^ cells was then calculated. For each image, the measured area fraction at day 17 was normalized by the corresponding pretreatment value in order to account for any variations in seeding density or outgrowth from well‐to‐well. Values obtained for the vehicle condition were averaged, and this average was subsequently used to normalized to treatment conditions where indicated.

#### 2.2.2 Quantification of TUNEL staining

After TUNEL staining, 12‐bit, 2048 × 2048 pixel images were acquired on a Zeiss LSM700 using a 0.3 NA 10X air objective (Zeiss, White Plains, NY, USA). For each pan‐cytokeratin‐positive tumor cell/cluster, the number of cells composing that cluster (based on DAPI signal) and the TUNEL status was tabulated manually. Cells were counted as TUNEL‐positive if they had strong (intensity > 1 × 10^3^), nonpunctate nuclear signal. Values reported are the percentage of TUNEL‐positive cells for each cluster analyzed, averaged for each condition over the total number of cells/clusters analyzed.

### 2.3 Western blotting

Bone marrow from the right femur was homogenized using 10 pulses of a Branson Sonifier 250. Lysate (40 μg) was then separated on a Tris‐Glycine 4–20% gel and transferred to a nitrocellulose membrane (Fisher, 1212590). Blots were blocked for 1 h at room temperature in 5% nonfat milk/PBS. Mouse monoclonal AKT (Cell Signaling Technologies, 2920, 1 : 1000) and rabbit monoclonal phospho‐AKT S478 (Cell Signaling Technologies, 4060, 1 : 1000) antibodies were probed as surrogate markers of Pi3K inhibition. Blots were incubated in primary antibody overnight at 4 °C and then washed six times over 30 min. Donkey anti‐rabbit 800 (Li‐Cor 926‐32213, 1 : 10 000) and donkey anti‐mouse 680LT (Li‐Cor 926‐68022, 1 : 10 000) were used as secondary antibodies and incubated at room temperature for 30 min. Blots were developed on a Li‐Cor Odyssey, and densitometry was performed on odyssey software v3.0 (LI‐COR Biosciences, Lincoln, NE, USA). Following development, blots were stripped and probed with a rabbit polyclonal antibody to the nuclear membrane protein Lamin A/C as a loading control (Santa Cruz Biotechnology, sc‐20681, 1 : 4000, Dallas, TX, USA) using donkey anti‐rabbit 800 as a secondary.

### 2.4 Animal studies

All animal work was performed in accordance with institutional, IACUC [specifically, Fred Hutchinson Cancer Research Center (Fred Hutch) protocol 51075] and AAALAS guidelines and ethical regulations.

#### 2.4.1 Pilot study

8‐ to 9‐week‐old female, Crl:NU(NCr)‐*Foxn1^nu^* mice (Charles River, Strain 490, Wilmington, MA, USA) were injected with either vehicle, 1 or 10 mg·kg^−1^ Gedatolisib (Pfizer, PF‐05212384, New York, NY, USA) via the tail vein. Gedatolisib vehicle consisted of 0.3% l‐lactic acid (Sigma, L6402) and 5% dextrose (Sigma, D9434) in endotoxin‐free ultrapure water (Millipore, TMS‐011‐A, Burlington, MA, USA) and was sterile‐filtered through a 0.2 μm PES filter. Gedatolisib was added to vehicle at 2 mg·mL^−1^, heated at 60 °C for 15–20 min, and then sterile‐filtered. Sterile PBS was used for balance, for a final injection volume of 100 μL. Animals were euthanized 1 or 2 h following injection.

#### 2.4.2 Harvesting bone marrow for protein lysate

Bone marrow was harvested from left and right femurs into 200 μL of 2% SDS/PBS containing complete protease inhibitor cocktail (Roche) and PhosSTOP phosphatase inhibitor cocktail (Roche). The ends of the femur were removed, and bone marrow was flushed into a 1.7‐mL Eppendorf tube using a 28 G insulin syringe loaded with lysis buffer. To ensure all bone marrow was collected, the bottom of a 0.6‐mL Eppendorf tube was trimmed, loaded with the femur, then placed into the appropriate 1.7‐mL Eppendorf tube. The tubes were then centrifuged at 16 837 x *g* for 30 s using an Eppendorf Centrifuge 5418 to ensure complete harvest of bone marrow. Samples were flash‐frozen in liquid nitrogen and stored at −80 °C until western blot analysis.

#### 2.4.3 Ovariectomy surgery

Five‐week‐old female, Crl:NU(NCr*)‐Foxn1^nu^* mice (Charles River, Strain 490) were ovariectomized by dual dorsal incisions to expose and dissect the ovaries without damaging the uterine horn. Firefly luciferase (ffluc), GFP expressing MCF‐7 was subsequently inoculated 14 days later via intracardiac (ic) injection.

#### 2.4.4 Intracardiac injection

At 7–8 weeks of age, anesthetized mice were positioned in dorsal recumbency on a VisualSonics Vevo 2100 Ultrasound Imaging System to guide injection of 1 × 10^5^ viable ffluc‐eGFP MCF‐7 or T4‐2 cells in 100 μL PBS. MCF‐7 were triturated upon harvest by passing 5 times through a 27‐G needle to obtain a single‐cell solution and drawn into a 1‐mL syringe mounted with a 27‐G needle for injections. Needle height and angle were adjusted on a stereotactic rig, and the needle was guided via ultrasound imaging into the left ventricle. The cell mixture was injected slowly only upon visualization of blood refluxing into the syringe. Bioluminescent imaging (BLI) was performed the following week using an IVIS Spectrum (Perkin Elmer) following intraperitoneal delivery of 100 μL d‐Luciferin (10 mg·mL^−1^, BioVision, Inc., Milpitas, CA, USA). The focal height was set to 0.7 cm. Mice with residual signal in their heart 1‐week postinjection were excluded from further study.

#### 2.4.5 Treatment

Five days postinjection, mice were primed with 10 mg·kg^−1^ Gedatolisib or vehicle and then treated weekly (days 7, 14, 21, and 28 postinjection) with inhibitor +/− doxorubicin/Adriamycin and cyclophosphamide (AC). Gedatolisib stocks were made up fresh prior to each injection and injected via the lateral tail vein. Note that the drug must be injected slowly to avoid toxicity. Injection of 100 μL over approximately 5 s or more was appropriate to prevent injection toxicity. Vehicle was stored at 4 °C and injected in the same manner. One hour following inhibitor or vehicle treatment, AC [2 mg·kg^−1^ Adriamycin/doxorubicin (Selleckchem S1208, in ddH20) and 60 mg·kg^−1^ cyclophosphamide (Selleckchem S2057)] or vehicle control (5% DMSO, 30% PEG300, 5% Tween in ddH20) were delivered intraperitoneally. Bioluminescent imaging (BLI) was performed weekly on an IVIS Spectrum (Perkin Elmer) following intraperitoneal delivery of 100 μL d‐Luciferin (10 mg·mL^−1^, BioVision, Inc.). The focal height was set to 0.7 cm.

To quantify DTCs remaining after treatment, one cohort of mice was euthanized on Day 29. A second cohort of mice was used to measure MFS over a prescribed course of 15 weeks.

#### 2.4.6 Quantification of bioluminescent flux in organs

Mice were injected with 100 μL d‐Luciferin 5–15 min prior to euthanasia. Following euthanasia, mice were perfused with 30 mL PBS via the left ventricle. Organs were harvested and imaged *ex vivo* on an IVIS spectrum with a focal height of 0.1 cm. Living Image software was used to quantify bioluminescent intensity of individual organs by manually tracing each organ to determine average bioluminescent signal. Background signal was acquired in two opposite locations, and then, the average background signal was used to normalize each organ’s signal to minimize variation between plates. Organs with high bioluminescent signal were removed, and adjacent images were re‐quantified to prevent bias from nonspecific signal. Normalized values were plotted on Prism and analyzed by two‐way ANOVA to determine statistically significant differences between treatment groups.

#### 2.4.7 Quantification of metastasis‐free survival

Mice were imaged weekly via BLI for a total of 15 weeks using the same methodology described above. Mice with a recurrent bioluminescent signal ≥ 10^4^ p·s^−1^·cm^−2^·sr^−1^ that increased over consecutive weeks were considered to have metastases.

#### 2.4.8 Bone marrow whole‐mounting and immunofluorescent staining

Bone marrow whole mounts were generated as detailed by Nombela‐Arrieta *et al*. [30]. OCT‐embedded femurs were shaved at 25 μm intervals on a Leica Cryostat CM3050 S (Leica Microsystems, Buffalo Grove, IL, USA) to expose bone marrow on one face, reversed, and sectioned to expose bone marrow on the opposite face. Sliced femurs were thawed from OCT, placed in a 1.7‐mL Eppendorf tube, washed twice with PBS, and blocked overnight (4 °C) in a solution containing sterile‐filtered (0.2 μm) 20% goat serum + 0.5% Triton X‐100 in PBS under constant, gentle agitation by circumferential rotation. Following blocking/permeabilization, bones were incubated for ~ 72 h in block containing the following antibodies: chicken anti‐GFP (Abcam ab13970, 1 : 1000) and rabbit anti‐pan‐laminin (Sigma L9393, 1 : 500). Bones were washed the following day a minimum of 6 times in PBS before washing overnight at 4 °C. Femurs were then submerged in blocking solution containing goat anti‐chicken 488 (Abcam ab150169, 1 : 500) and goat anti‐rabbit 568 (Invitrogen A11011, 1 : 500) secondary antibodies and DAPI (2 μg·mL^−1^). Femurs were washed again the following day in PBS and finally placed into a solution of PBS + 0.02% sodium azide to preserve the bone prior to imaging.

For imaging, whole mounts were generated by gently placing femurs in aqueous mounting medium (Fluoromount‐G, Southern Biotech, Birmingham, AL, USA) in a 3.5 cm plate containing a No 1.5 coverslip insert (MatTek Corporation, P35G‐1.5‐20‐C, Ashland, MD, USA), and covering the femur with a 18 × 18 mm coverslip in order to press the marrow flush against the imaging window. Tile scans were generated by placing this culture dish on a Zeiss LSM700 and scanning the entire bone using three laser lines and a 0.17 NA 2.5× Fluar objective to quantify DTC number. Images of individual DTCs were acquired using a 0.55 NA 20× air objective. The number of DTCs was normalized to the corresponding bone marrow area and the values for each side of bone were averaged.

#### 2.4.9 Lung clearing via the Ce3D method and staining GFP^+^ DTCs

After euthanasia, the left lobe of the lung was harvested and fixed in 1% PFA/PBS overnight, rocking at 4 °C. Lungs were blocked in 1.5 mL of 1% normal mouse serum (MP Biomedicals, Santa Ana, CA, USA), 1% BSA (Sigma), 0.3% Triton X‐100 (Sigma) in PBS for 8 h at 37 °C in a shaking incubator. Chicken anti‐GFP antibody (Abcam ab13970, 1 : 500) was added to blocking solution, and lungs were incubated in primary antibody solution for 72 h while shaking at 37 °C. Lungs were washed for 24 h in 0.2% Triton X‐100 and 0.5% 1‐thioglycerol (Sigma) in PBS while shaking at 37 °C. Lungs were submerged in 1.5 mL block solution containing goat anti‐chicken 488 (Abcam, ab150169, 1 : 250) and shaken for another 48 h at 37 °C. Wash was repeated for another 24 h.

After staining, lungs were cleared using Ce3D [31] clearing solution (40% N‐methylacetamide (Sigma) v/v, 86% w/v Histodenz (Sigma), 0.1% Triton X‐100 and 0.5% 1‐thioglycerol in PBS). Lungs were covered and incubated in 1 mL Ce3D clearing solution on a circumferential rotor for 72 h (RT). Ce3D clearing solution was replaced and lungs were incubated for another 72 h. Cleared lungs were stored in Ce3D clearing solution at room temperature until imaged. Imaging to quantify DTCs was performed manually in single‐blinded fashion on a Zeiss AXIO Zoom.V16 with the aperture set to 65%. Lungs were scanned at 37.5X, and structures of interest were confirmed to be cellular in nature at 112X. A cleared lung from an uninoculated mouse was used as a control; no DTCs or DTC‐like structures were observed in this tissue.

### 2.5 Statistics and reproducibility

Statistical analyses were conducted with graphpad prism 7 software. Data that were distributed normally were compared via unpaired, two‐tailed *t*‐test (if only two conditions were tested) or via one‐ or two‐way ANOVA (for experiments containing three or more conditions). Post‐testing to correct for multiple comparisons was chosen based on whether a decision was made a priori to compare conditions to vehicle (Dunnett’s) or to compare all treatment groups with each other (Tukey’s). Data that were not distributed normally were tested via Kruskal–Wallis and Dunn’s post‐test to correct for multiple comparisons. Please refer to figure legends for individual *n*‐ and *P*‐values, and the specific statistical test(s) employed. Unless noted otherwise, data are reported such that the centerline represents the mean, and error bars represent the SEM.

## 3 Results

### 3.1 Src and PI3K emerge as putative regulators of chemoresistance in dormant tumor cells in organotypic culture

**Fig. 1:**
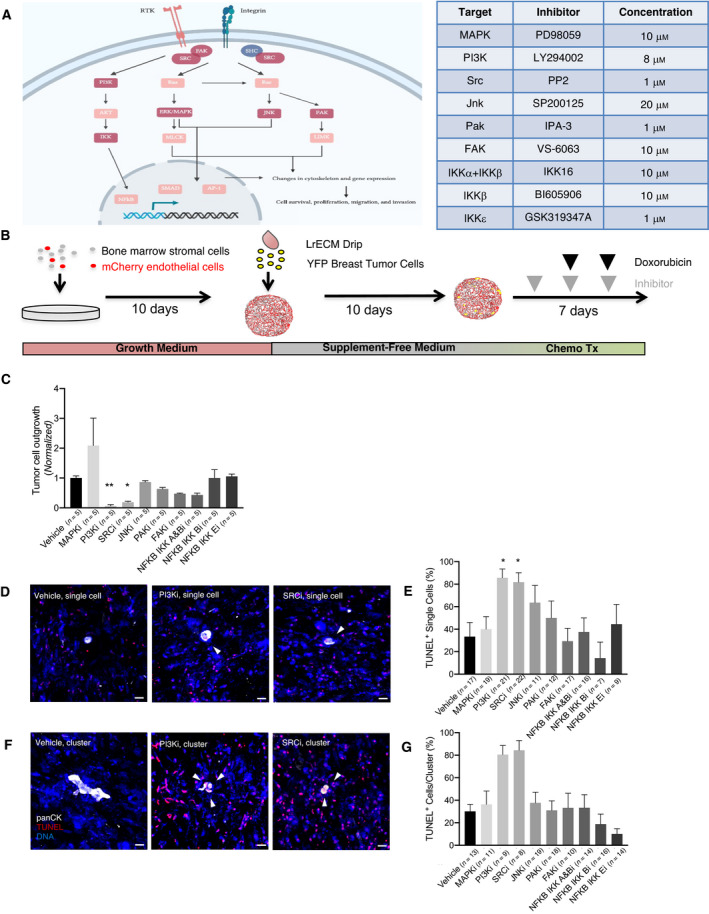
Probing signaling downstream of integrin‐β1 suggests PI3K and Src as therapeutic targets. (A) Canonical integrin signaling pathways, with key nodes highlighted in dark red. Signaling pathway adapted from reference [18]. Inhibitors targeting indicated nodes are listed in the accompanying table, along with the doses used for *in vitro* treatment in our study. (B) Schematic of bone marrow microvascular niche (bm‐MVN) culture experiments to determine the effect of targeting downstream integrin signaling. HMT‐3522‐T4‐2s were seeded onto bm‐MVNs and dosed with small molecule inhibitors. (C) PI3K inhibition significantly decreased tumor cell outgrowth (*P* = 0.0024), as did Src inhibition (*P* = 0.0138). Following completion of the experiment, cultures were TUNEL‐stained to determine tumor cell apoptosis. A Kruskal–Wallis test comparing data from five replicates was used here. Outgrowth is normalized to vehicle. Error bars represent SEM. (D) Representative images of TUNEL‐stained single tumor cells on bm‐MVNs treated with DMSO (vehicle), PI3K inhibitor (LY294002), or SRC inhibitor (PP2) followed by doxorubicin. Scale bar = 20 μm. White arrows indicate TUNEL‐positive apoptotic tumor cells. (E) Quantification of the percentage of TUNEL‐positive single tumor cells. PI3K and SRC inhibition significantly increased apoptosis in single cells (*P* = 0.018 and *P* = 0.035, respectively) when compared to vehicle via Kruskal–Wallis test. *n* = 7–22 single cells analyzed per condition. Error bars represent SEM. (F) Representative images of TUNEL‐stained 2–4 cell clusters treated with DMSO, PI3K, or SRC inhibitors. (G) Quantification of the percentage of TUNEL‐positive clusters of tumor cells. Kruskal–Wallis test shows PI3K inhibition increases apoptosis in 2–4 cell clusters (*P* = 0.006), as does SRC inhibition (*P* = 0.005), when compared to vehicle. *n* = 8–19 clusters analyzed per condition. Error bars represent SEM.

The intracellular signaling cascade downstream of integrins comprises key signaling hubs such as PI3K/Akt, MAPK/ERK, and JNK, which are activated by upstream SRC and/or FAK, and can affect survival via NFκB signaling (Fig. 1A) [18]. Small molecule inhibitors have been developed and implemented clinically for most of these signaling nodes, making them attractive potential target to sensitize DTCs to chemotherapy.

We tested nine small molecule inhibitors against known downstream targets of integrin‐β1 (Fig. 1A). Based on prior work demonstrating that disseminated breast tumor cells occupy the PVN, where they are steered into quiescence while also conferred with resistance to genotoxic therapy [5], we conducted chemosensitization experiments in organotypic models resembling the bone marrow’s perivascular microenvironment [5, 12]. Briefly, primary human bone marrow stromal cells (mesenchymal stem cells) were co‐cultured with endothelial cells (HUVEC) to create an organotypic model of the bone marrow’s microvascular niche, which was later seeded sparsely with breast tumor cells. The majority of breast tumor cells [in this case triple‐negative yellow fluorescent protein (YFP)‐expressing‐HMT‐3522‐T4‐2 cells [28, 32], which cluster molecularly as basal [33]] become quiescent when seeded in culture in serum‐ and cytokine‐free conditions in these organotypic models [12], allowing us to assess viability of dormant cells following treatment. We ‘primed’ bone marrow microvascular niche (bm‐MVN) cultures with small molecule inhibitors to allow for initial sensitization of cells. Subsequent treatments occurred 2 and 5 days following priming treatment and consisted of inhibitor followed by doxorubicin 1 h later (Fig. 1B). Because the majority of cytokeratin‐positive DTCs found in patient bone marrow aspirates are quiescent single cells, and rarely clusters exceeding four cells [16, 34], we assessed viability of single cells and 2–4 breast tumor cell clusters via TUNEL staining. Vehicle (DMSO)‐primed cultures treated subsequently with 2500 nm doxorubicin [5] resulted in death of one‐third of single T4‐2 cells, and 30.1% of T4‐2 cell clusters (Fig. 1D–G), consistent with previous results [5]. Inhibition of key integrin‐β1 signaling effectors FAK (with VS‐6063), MAPK (with PD98059), JNK (with SP200125), PAK (with IPA‐3), as well the NFκb de‐repressor IKK kinase complex (with NFKB IKK A&Bi, NFKB IKK Bi, and NFKB IKK Ei, three different but strategically overlapping IKK subunit inhibitors) had no effect on T4‐2 cell burden on bm‐MVNs in the absence of doxorubicin treatment (Fig. 1C). Nor did any of these inhibitors induce apoptosis of single breast tumor cells or breast tumor cell clusters in combination with doxorubicin any more than doxorubicin on its own (Fig. 1D–G). On the other hand, the inhibition of Src (with PP2) and of PI3K (with LY294002) induced apoptosis of more than 80% of T4‐2 single cell and 2–4 cell clusters (Fig. 1D–G), implicating both molecules as potent regulators of chemosensitivity in dormant tumor cells.

### 3.2 The PI3K/mTOR inhibitor Gedatolisib chemosensitizes dormant tumor cells to doxorubicin in organotypic culture

Following identification of PI3K and Src as potential key regulators of chemoresistance in dormant T4‐2 cells, we tested the dose‐dependent response of two breast cancer cell lines—TNBC T4‐2 cells and ER^+^ MCF‐7 cells—to the Src inhibitor Bosutinib (SKI‐606) and PI3K/mTOR inhibitor Gedatolisib (PF‐05212384). Both compounds have advanced to human trials [35, 36, 37] and were tested because of our desire to move beyond tool compounds for eventual preclinical and clinical studies. In combination with doxorubicin, Bosutinib depleted T4‐2 cell burden on bm‐MVNs only at high doses and had no impact on MCF‐7 at any of the doses tested (data not shown). Because of this lack of efficacy across models of both luminal and basal breast cancer cell dormancy, we focused our efforts on Gedatolisib.

**Fig. 2:**
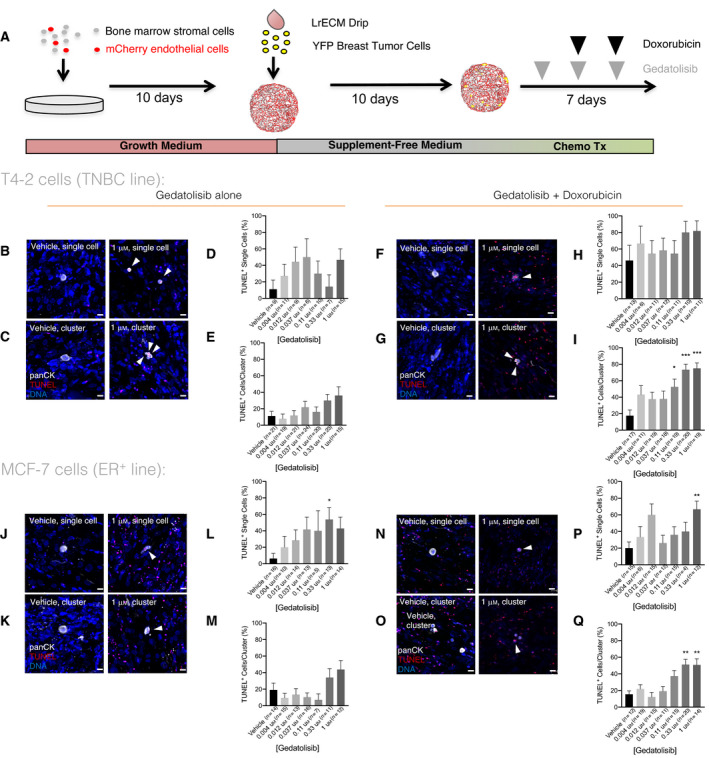
The PI3K inhibitor Gedatolisib chemosensitizes dormant breast cancer tumor cells in organotypic culture. (A) Schematic of bm‐MVN experimental design. (B,C) Representative images of (B) a single T4‐2 cell and (C) a cluster of T4‐2 cells treated with either vehicle or 1 μm Gedatolisib, fixed and TUNEL‐stained at the experimental endpoint indicated in Fig. 1B. Scale bar = 20 μm. White arrows illustrate TUNEL‐positive tumor cells. (D) Analysis of TUNEL‐positive single tumor cells treated with escalating doses of Gedatolisib. *n* = 6–15 single cells analyzed per condition. Error bars represent SEM. (E) Analysis of TUNEL‐positive T4‐2 cell clusters treated with escalating doses of Gedatolisib. *n* = 15–24 cells/clusters analyzed per condition. Error bars represent SEM. (F,G) Representative images of (F) single and (G) clustered T4‐2 cells treated either with doxorubicin (2500 nm) alone or in combination with 1 μm Gedatolisib. (H,I) Analysis of TUNEL‐positive (H) single and (I) 2–4 cell T4‐2 cell clusters treated with doxorubicin (2500 nm) escalating doses of Gedatolisib. *n* = 6–20 cells/clusters analyzed per condition. Gedatolisib significantly increases the average T4‐2 cluster death at 0.11 μm (53% TUNEL^+^, *P* = 0.0434), 0.33 μm (73% TUNEL^+^, *P* < 0.0001), and 1 μm (75% TUNEL^+^, *P* < 0.0001). Kruskal–Wallis tests were used to determine statistical significance, relative to vehicle. Error bars represent SEM. (J,K) Representative images of (J) single and (K) a cluster of MCF‐7 cells treated with either vehicle or 1 μm Gedatolisib, fixed and TUNEL‐stained at the experiment’s end. The scale bar denotes 20 μm and the white arrows illustrate TUNEL‐positive cells. l‐m) Analysis of TUNEL‐positive (L) single and (M) 2–4 cell MCF‐7 cell clusters treated with escalating doses of Gedatolisib. *P = 0.004 via Kruskal–Wallis test, with respect to vehicle; *n* = 5–16 clusters analyzed per condition. Error bars represent SEM. (N,O) Representative images of (N) single and (O) a cluster of MCF‐7 cells treated either with doxorubicin (2500 nm) or 1 μm Gedatolisib and doxorubicin. (*P*) Analysis of TUNEL‐positive single MCF‐7 cells treated with doxorubicin (2500 nm) escalating doses of Gedatolisib. **P = 0.003, as determined by Kruskal–Wallis test; *n* = 15–30 single cells were analyzed per condition. Error bars represent SEM. (Q) Analysis of TUNEL‐positive 2–4 cell MCF‐7 cell clusters. The two highest doses of Gedatolisib, in combination with doxorubicin, significantly increased apoptosis of MCF‐7 clusters (1 μm *P* = 0.0049; 0.33 μm *P* = 0.001) when compared to vehicle‐treated cultures via Kruskal–Wallis test; *n* = 15–40 clusters were analyzed per condition. Error bars represent SEM.

In the absence of doxorubicin, Gedatolisib only modestly induced apoptosis of individual T4‐2 cells on bm‐MVNs (Fig. 2A,B,D). Its effect on 2–4 cell clusters trended toward statistical significance (*P* = 0.13 at a dose of 1 μm; Fig. 2C,E). On its own, doxorubicin induced apoptosis of 46.2% of single T4‐2 cells, whereas 80.0% and 81.8% of single T4‐2 cells were TUNEL‐positive after priming with the two highest doses of Gedatolisib (Fig. 2F,H). Gedatolisib also exhibited dose dependence in combination with doxorubicin on clusters of T4‐2 cells; 17.6% of clusters contained TUNEL‐positive cells with doxorubicin alone, whereas 75.0% contained apoptotic cells when primed with 1 μm Gedatolisib (Fig. 2G,I). The impact of Gedatolisib treatment in a model of luminal B breast cancer cell dormancy largely mimicked what was observed with basal T4‐2 cells. In single MCF‐7 cells, Gedatolisib efficacy peaked at 0.33 μm, where it induced apoptosis of 53.8% of cells (Fig. 2J,L). Its effect on 2–4 cell clusters was more modest and did not approach statistical significance (Fig. 2K,M). However, Gedatolisib effectively chemosensitized single MCF‐7 cells and 2–4 cell clusters at high doses and significantly enhanced response to doxorubicin, as judged by TUNEL staining (Fig 2N–Q). Doxorubicin on its own resulted in death of 20.0% of single cells, whereas priming with 1 μm Gedatolisib induced apoptosis of two‐thirds of single cells (Fig. 2N,P). MCF‐7 clusters were sensitized to a similar degree; apoptotic cells were detected in 15.4% of MCF‐7 clusters treated with doxorubicin alone, whereas priming with 0.33 or 1 μm Gedatolisib induced apoptosis in just over half of the clusters quantified (51.2% and 50.8%, respectively; Fig. 2O,Q). Taken together, these data indicate that although Gedatolisib is mildly effective in killing dormant tumor cells on its own, the drug synergizes with genotoxic therapy (doxorubicin, specifically) to induce apoptosis of quiescent breast tumor cells in organotypic culture. Thus, Gedatolisib is a potentially viable priming agent to sensitize DTCs to chemotherapy for the purposes of preventing breast cancer metastasis.

### 3.3 Gedatolisib does not significantly decrease metastatic burden or delay onset of metastatic disease in a mouse model of TNBC

Previous studies demonstrated Gedatolisib efficacy against multiple solid tumor types, especially in combination with other therapies [38, 39, 40]. However, these studies predominantly tested drug activity on ectopically implanted tumors (e.g., subcutaneous) and used stasis as an indicator of efficacy [38, 40]. Based in part on these data, the GLACIER clinical trial (NCT03400254; now withdrawn) aimed to uncover whether Gedatolisib would eliminate DTCs in TNBC patients within 5 years of initial treatment. Given the demonstrated efficacy of Gedatolisib against quiescent breast tumor cells in organotypic culture, we designed a preclinical study to (a) determine whether PI3K/mTOR is a critical node downstream of integrin‐β1 *in vivo* and (b) coincide with the GLACIER trial to determine the predictive capacity of these preclinical models.

**Fig. 3:**
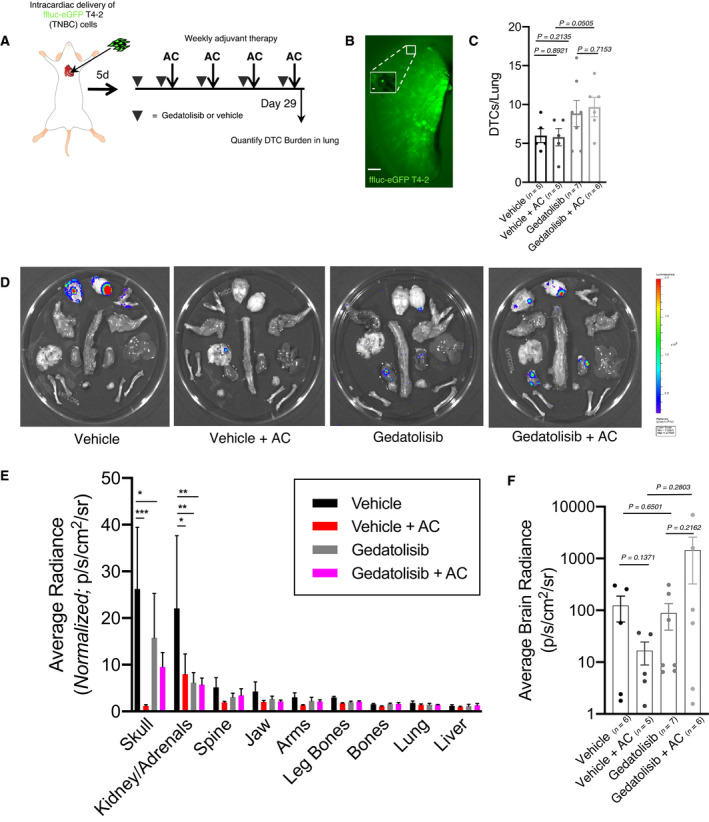
Gedatolisib does not prevent or decrease metastatic burden in a TNBC preclinical model of breast cancer. (A) Schematic of preclinical trial design. (B) Representative image of a Ce3D‐cleared lung stained with anti‐GFP antibody and imaged at 7×. Scale bar = 1 mm. A single DTC (112×) is shown in the insert. Scale bar = 20 μm. (C) Quantification of DTCs present in cleared lungs. The listed *P*‐values represent comparisons of the indicated conditions via unpaired, two‐tailed *t*‐test. Error bars represent SEM. (D) Representative images of *ex vivo* bioluminescent signal detected in organs from sacrificed mice as in Fig. 3A. (E) Quantification of bioluminescent signal present in organs of mice euthanized at endpoint. Gedatolisib treatment, with or without chemotherapy, minimally reduces metastatic burden in mice. Vehicle‐treated mice experience the highest average metastatic burden across organs, whereas mice treated with chemotherapy have significantly decreased tumor burden in the skull (*P* = 0.009) and the kidney/adrenals (*P* = 0.0131). Gedatolisib significantly reduces metastatic burden in the kidney/adrenals (*P* = 0.016) and modestly reduces metastatic burden in most tissues compared to vehicle alone. Note that the tissues are arranged from highest average signal (skull) to lowest average signal (liver). Two‐way ANOVA was used to determine statistical significance. Error bars represent SEM. (F) Quantification of bioluminescent signal present in brains of mice euthanized at endpoint. Gedatolisib treatment increases the average intensity of brain metastasis in mice treated with chemotherapy (average_flux Gedatolisib + AC_ = 1451 p·s^−1^·cm^−2^·sr^−1^, average_flux vehicle + AC_ = 17 p·s^−1^·cm^−2^·sr^−1^). Unpaired, two‐tailed *t*‐tests were used to determine statistical significance. For each analysis presented in (C), (E), and (F), organs were analyzed from *n* = 5 mice for vehicle and vehicle + AC arm, *n* = 7 mice for Gedatolisib arm, and *n* = 6 mice for Gedatolisib + AC treatment arm. Error bars represent SEM.

Gedatolisib (10 mg·kg^−1^) optimally decreased pAkt^S478^, a surrogate marker of PI3K activation [40], in bone marrow cells 1 h after intravenous injection (Fig. S1). A substantive effect was not observed with a log‐scale reduction in dose (Fig. S1). This suggested that mirroring the regime trialed in culture (Fig. 2; i.e., infusing 10 mg·kg^−1^ Gedatolisib and then delivering genotoxic therapy 1 h later) could sensitize DTCs in bone marrow and elsewhere to chemotherapy. Therefore, we set up a multi‐armed preclinical study to test this hypothesis. Athymic nude mice were injected with ffluc‐eGFP T4‐2 cells via the intracardiac (ic) route to ensure metastatic dissemination to lung, brain, liver, and bones (the primary metastatic sites of breast cancer), primed with 10 mg·kg^−1^ Gedatolisib or the corresponding volume of vehicle 5 days later. Starting 7 days after ic injection, mice were treated with Gedatolisib or corresponding vehicle followed 1 h later by 2 mg·kg^−1^ doxorubicin/Adriamycin and 60 mg·kg^−1^ cyclophosphamide (AC) or corresponding vehicle (Fig. 3A). Mice were treated weekly over 4 weeks to approximate dose‐dense chemotherapeutic regimens prescribed to patients with invasive breast cancer [41]. The day after the last treatment dose, mice were euthanized, and tissues were harvested to quantify DTC burden (number of GFP^+^ DTCs) and overall metastatic burden (luciferase signal from whole organs). Gedatolisib alone did not yield an obvious advantage in decreasing or preventing metastatic disease compared to vehicle‐treated mice. Nor did Gedatolisib augment dose‐dense AC therapy in a manner consistent with alleviation of disease burden (Fig. 3B–F). Vehicle‐treated mice had a roughly equivalent number of DTCs in the left lobe of the lung compared to mice treated with AC, Gedatolisib, or Gedatolisib + AC (Fig. 3B–C). T4‐2 cells delivered to athymic nude mice via intracardiac injection tend to form metastases in their skull, brain, and adrenal glands. Regimens containing dose‐dense AC yielded a significant and substantial decrease in average luciferase signal in the skull and adrenal glands (Fig. 3D–E). Signal in all other tissues assessed, including brain, was unaffected (Fig. 3E–F). On its own, Gedatolisib did result in a significant decrease in luciferase signal emitted from the kidneys and adrenal glands (Fig. 3E). However, priming with Gedatolisib did not enhance the efficacy of AC. In fact, the two mice with the highest brain luciferase signal in this study received the PI3K/mTOR inhibitor in addition to dose‐dense AC regimen (Fig. 3E). These data demonstrate that Gedatolisib, alone or in combination with AC, does not reliably or significantly decrease metastatic burden or eradicate DTCs in a preclinical model of TNBC metastasis. On the contrary, dose‐dense chemotherapy offers the best treatment option across metrics measured, and addition of Gedatolisib skews some of these metrics in the wrong direction.

### 3.4 Gedatolisib does not eliminate DTCs or prevent metastasis in a mouse model of ER^+^ breast cancer metastasis

More than half of women who experience distant, metastatic recurrence of ER^+^ breast cancer recur after completing 5 years of endocrine therapy [2]. Therefore, these patients may benefit most from a DTC‐targeted therapy that eliminates the source of metastasis.

**Fig. 4:**
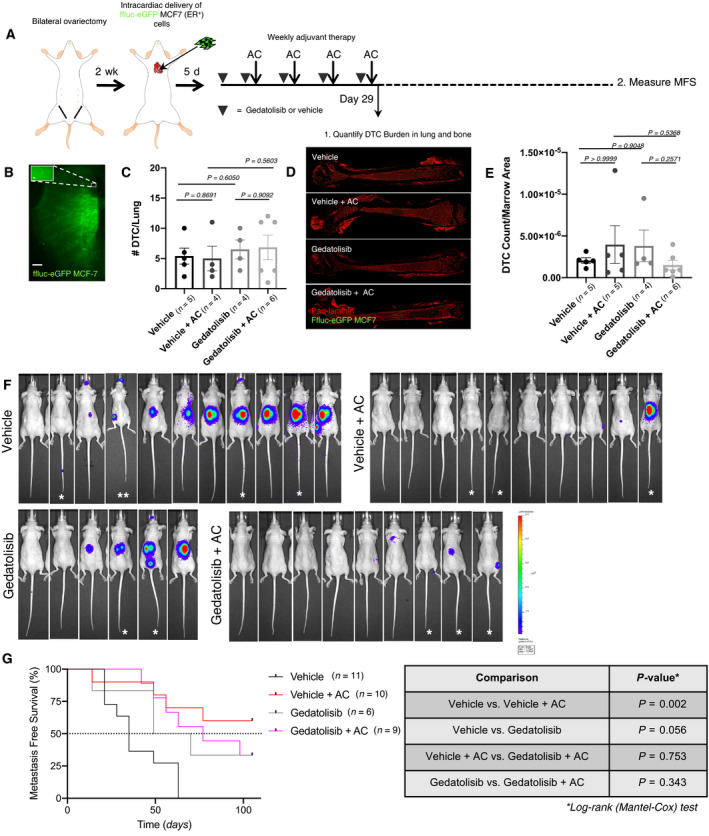
Gedatolisib does not prevent, decrease or delay metastatic disease in an ER^+^ preclinical model of breast cancer. (A). Schematic of preclinical trial design. (B) Representative image of a DTC in a Ce3D‐cleared lung, stained for GFP. Scale bar = 1 mm. A single DTC (112×) is shown in the insert. Scale bar = 20 μm. (C) Analysis of DTC burden measured in Ce3D‐cleared lungs (analyzed statistically via unpaired two‐tailed *t*‐test). Treatment with Gedatolisib and/or chemotherapy does not significantly alter DTC presence in the lung. Error bars represent SEM. (D) Representative images of whole‐mounted femurs stained with GFP and pan‐laminin antibodies. (E) Analysis of DTC burden in whole‐mounted femurs, normalized by area (μm^2^) of marrow analyzed. Data were analyzed statistically via unpaired two‐tailed *t*‐test. There were no significant changes in DTC burden in the bone marrow of mice across treatment groups. For cohort 1, organs were analyzed from *n* = 5 mice (vehicle), *n* = 4 mice (vehicle + AC), *n* = 4 mice (Gedatolisib only), and *n* = 6 mice (Gedatolisib + AC). Error bars represent SEM. (F) Bioluminescent images of mice 15 weeks after tumor cell inoculation. Bones (spine, hind bones, or vertebral tail bones) and adrenal glands were the most common site of metastasis. Mice that did not receive chemotherapy harbored metastases at multiple sites. Group sizes were as follows: *n* = 11 mice (vehicle), *n* = 10 mice (vehicle + AC), *n* = 6 mice (Gedatolisib), and *n* = 9 mice (Gedatolisib + AC). * animal was euthanized at week 14 postinjection. ** animal was euthanized at week 4 postinjection. (G) Kaplan–Meier plot of metastasis‐free survival. *P‐*values for all relevant comparisons listed in the accompanying table obtained via Log‐rank (Mantel‐Cox) test. Chemotherapy significantly improved metastasis‐free survival (*P* = 0.002), compared to vehicle‐treated mice. Gedatolisib treatment did not offer survival advantage compared to other treatment groups.

To model estrogen suppression, we ovariectomized female, athymic nude mice. Two weeks later, these mice were injected via the left ventricle with ffluc‐eGFP MCF‐7 cells. We then followed an identical treatment plan as used in experiments with the TNBC preclinical model (Fig. 3A). In addition to a first cohort of mice that was sacrificed following cessation of treatment, a second cohort remained on study for 15 weeks to track metastatic progression via intravital luciferase imaging (Fig. 4A). In the first cohort, no treatment (Gedatolisib, AC, or Gedatolisib + AC) depleted the number of DTCs present in the lung (Fig. 4B–C) or in the bone marrow (Fig. 4D–E), as assessed in cleared left lung lobes or whole‐mounted femurs of said mice. Our previous study revealed that a substantial depletion of lung and bone DTCs in this model corresponded to a substantial enhancement in MFS [5]. In the present study, only dose‐dense AC significantly improved MFS (Fig. 4F–G). Although Gedatolisib treatment on its own decreased penetrance of metastases, it did not significantly delay time to first metastasis, nor significantly improve MFS compare to vehicle‐treated mice (Fig. 4F–G). Paradoxically, the addition of Gedatolisib to dose‐dense AC increased the fraction of mice that developed metastases over time.

Taken together, these data indicate that Gedatolisib, alone or in combination with chemotherapy, is not effective in targeting, preventing, or delaying metastasis in preclinical setting. Gedatolisib has no significant effect on reducing DTC burden in both TNBC and ER^+^ models, and addition of the drug to chemotherapy does not enhance chemotherapeutic efficacy. Thus, despite showing promise in an organotypic setting, Gedatolisib has minimal efficacy in a preclinical setting with therapeutically relevant endpoints.

## 4 Discussion

Selective targeting of dormant DTCs holds promise as an approach for metastasis prevention [11, 42, 43]. One way to achieve such targeting is to sensitize dormant cells to cytotoxic therapy. Previously, we found that disseminated breast tumor cells located within the bone marrow’s vascular niche are protected from chemotherapy by endothelial cell‐derived ligands—independent of DTC cell cycle status—and that disrupting these interactions by targeting DTC integrin‐β1 sensitized DTCs to chemotherapy [5]. Given the dearth of clinically approved integrin‐β1 targeting antibodies and the potential impact of this approach in clinical settings, we sought to identify signaling hubs downstream of integrin‐β1 that could be targeted more readily by small molecule inhibitors. Our studies in organotypic culture identified PI3K as a potential node of integrin‐β1‐mediated chemoprotection. Studies with a clinically approved PI3K/mTOR inhibitor (Gedatolisib) in the same model showed marginal efficacy against quiescent breast tumor cells on its own, but substantial synergy with doxorubicin. However, efficacy in organotypic culture did not translate to preclinical models of TNBC or ER^+^ breast cancer. Gedatolisib, alone or in combination with dose‐dense AC, did not effectively deplete DTCs from lung or bone marrow, prevent metastasis or prolong MFS.

The clinical application of therapeutic regimens that target PI3K in breast cancer has long held allure. This can be attributed primarily to three factors. One is the prevalence of activating mutations in the catalytic subunit of PI3K, PIK3CA, in breast cancer. PIK3CA is the most frequently mutated gene across ductal and lobular breast carcinomas [44]. Another is the functional relevance of PI3K in breast cancer initiation. The most common PIK3CA activating mutation, H1047R, is sufficient to drive mammary tumorigenesis in mice [45] and is a potent ingredient in transformation of human mammary epithelial cells [46, 47]. Finally, hyperactivation of the PI3K pathway is linked to resistance to endocrine therapy in ER^+^ breast cancer cell lines [48] and to anti‐HER2 therapy in transgenic mice [49]. Ostensibly, these data were the principal motivation behind the GLACIER study, which aimed to test whether Gedatolisib, in combination with the anti‐malaria drug hydroxychloroquine, showed any evidence of DTC eradication in patients with TNBC or with HER2‐amplified breast cancer. Although this study was withdrawn due to a ‘business decision regarding the study drug,’ (Source: https://clinicaltrials.gov/ct2/show/NCT03400254) our results cast doubt on the ability of Gedatolisib specifically‐‐ and PI3K/mTOR inhibitors more generally—as secondary prevention agents.

This evidence raises questions on the kind of data that should be used to plan future trials aimed at metastasis prevention. Our contention is that prior work assessing PI3K inhibition in breast cancer, including but not limited to Gedatolisib, predominantly gauged efficacy based on achieving stasis of orthotopically implanted primary tumors or of spontaneous tumors in genetically engineered mouse models [38, 40, 49]. There are substantial differences in the biology of DTCs and primary tumors related to cell cycle status and microenvironmental signaling [11, 50, 51]. In humans, this extends to intrinsic properties of the cells themselves, since DTCs have less genomic aberrations than matched primary tumors [52, 53, 54, 55]. There is also a substantive difference in treatment paradigm, whether it be daily application of a kinase inhibitor to keep a tumor at bay, or intermittent treatment and release in order to prevent eventual recurrence. Based on these factors alone, it is critical to model therapy in context. In doing so, our work suggests that PI3K inhibition is not sufficient to target DTCs or prevent metastasis, with or without dose‐dense chemotherapy.

It is important to note that in our hands organotypic culture models predicted efficacy of Gedatolisib in the bone marrow microenvironment and clearly missed the mark. One possible explanation is that metastasis‐initiating capacity of residual cells is not assessed in this assay. Ample evidence shows that chemotherapy enriches for breast cancer cells with stem‐like properties [56, 57, 58]. Perhaps integrin‐β1 blockade facilitates chemotherapeutic eradication of breast tumor cells with metastasis‐initiating capacity, whereas PI3K/mTOR inhibition does not. Or, any of a myriad of downstream effectors [18] could compensate for loss of PI3K activity *in vivo*. For example, our preliminary data derived from analysis of reverse‐phase protein array (S‐Y Lee *et al*., unpublished) suggest that FAK is activated in dormant breast cancer cells—specifically upon treatment with Gedatolisib and doxorubicin. Therefore, rewiring survival signaling through FAK may mediate DTC survival in response to these particular cellular stresses, which would not be wholly unexpected [59]. But even if combining a FAK inhibitor with Gedatolisib achieved sensitization to doxorubicin *in vivo* at levels that approach those achieved with integrin‐β1 inhibition [5], working out the sequencing and mitigation of potential toxicities for three drugs is more complex than with two. Thus in our view, effort would be better invested in the clinical development of an integrin‐β1 inhibitor, since the efficacy of targeting this upstream mediator of DTC survival is substantiated already [5].

A final point regards the observation that adding Gedatolisib to dose‐dense AC resulted in a trend toward more extensive brain metastases than dose‐dense AC alone in a TNBC model. It is tempting to speculate that systemic hyperinsulinemia induced by PI3K inhibition, a recently described phenomenon [60], might be playing a role. If PI3K inhibition resulted in a statistically and biologically significant increase in brain metastases in large experimental cohorts and in additional TNBC models, it would be interesting to determine whether such an effect could be mitigated by intervening in the acute glucose‐insulin feedback triggered by PI3K inhibition.

## 5 Conclusions

The preclinical data presented here suggest that a trial relying on PIK3CA inhibition to target DTCs and prevent breast cancer metastasis would not meet the surrogate endpoint of DTC clearance or the functional endpoint of enhanced MFS. These data affirm the need to identify druggable targets of DTC survival in the presence or absence of chemotherapy. They also re‐affirm the need for preclinical models that measure clinically relevant endpoints, similar to those one wishes to achieve with a targeted patient population.

## Conflict of interest

The authors declare no conflict of interest.

### Peer Review

The peer review history for this article is available at https://publons.com/publon/10.1002/1878‐0261.13031.

## Author contributions

RES, DL, MJB, and CMG designed experiments. RES, JD, S‐YL, and LP performed experiments. RES, S‐YL, IM, and SL acquired and analyzed data. RES and CMG wrote the manuscript. All authors read the manuscript and provided critical feedback.

## Supporting information


**Fig. S1**. Intravenous administration of Gedatolisib results in suppression of AKT activation in bone marrow. (A) Western blots probing for p‐Akt and total Akt in bone marrow cells 1‐h after mice were injected via tail vein with Gedatolisib vehicle (0.3% lactic acid, 5% dextrose), 1 mg·kg^−1^ Gedatolisib or 10 mg·kg^−1^ Gedatolisib. (B) Analysis of band intensities (pAKT normalized by total AKT) presented for each treatment condition. *P = 0.012 when compared to vehicle by ANOVA and Dunnett’s post‐test. *n* = 3 femurs from three individual mice analyzed per treatment condition. Error bars represent SEM. (C) Western blots probing for p‐Akt and Total Akt in bone marrow cells two‐hours after mice were injected via tail vein with vehicle, 1 mg·kg^−1^ Gedatolisib or 10 mg·kg^−1^ Gedatolisib. (D) Analysis of band intensities (pAKT normalized by AKT) presented for each treatment condition. *P = 0.022 when compared to vehicle by ANOVA and Dunnett’s post‐test. Femurs from *n* = 3 mice analyzed per treatment condition. Error bars represent SEM.

## Data Availability

All raw data are will be shared by the corresponding author upon request.
