# Supplementary material for: The PI3K/mTOR inhibitor Gedatolisib eliminates dormant breast cancer cells in organotypic culture, but fails to prevent metastasis in preclinical settings
Source: Mol Oncol. 2021 Jun 25;16(1):130–47. doi: 10.1002/1878-0261.13031 (PMC8732345; doi:10.1002/1878-0261.13031)

Supplemental Figure 1: Gedatolisib significantly suppresses AKT activation in the bone marrow

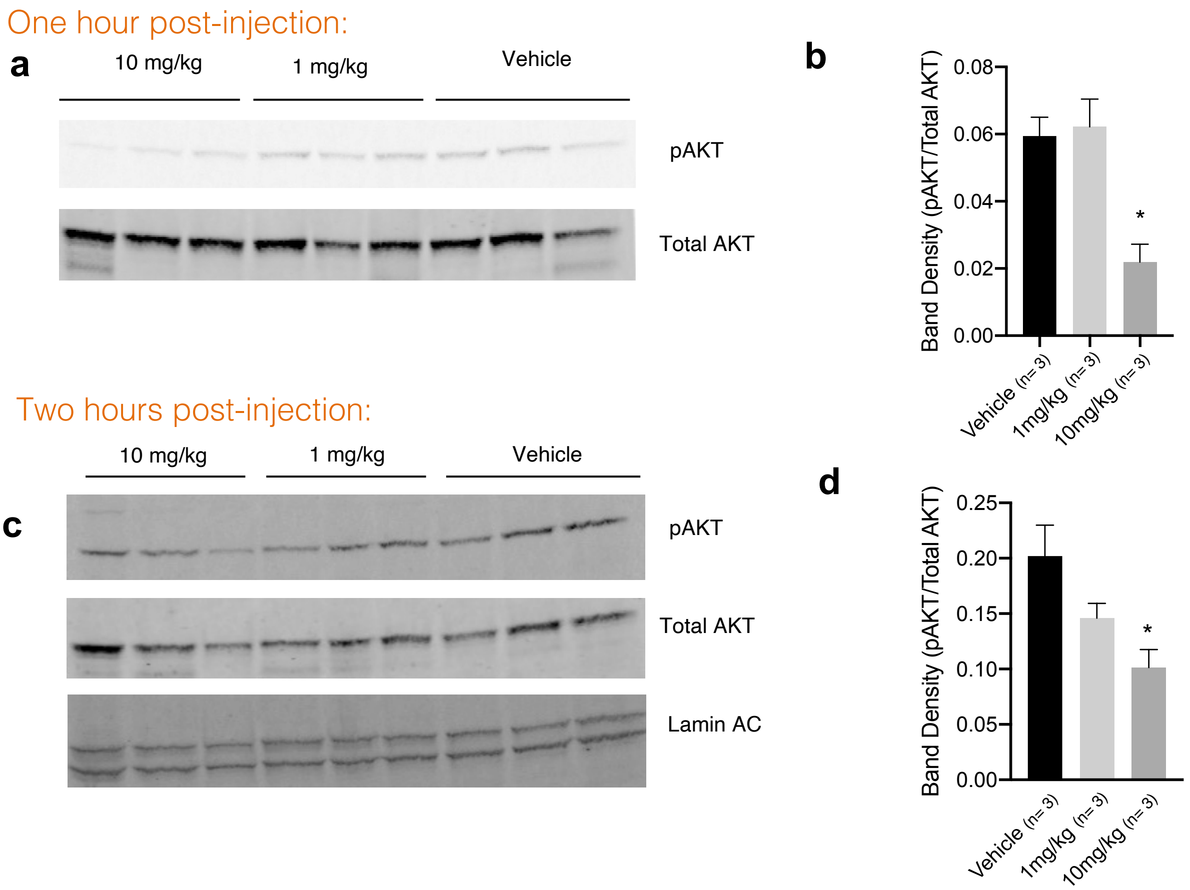

Supplement: Supplementary file 1 — Fig. S1. Intravenous administration of Gedatolisib results in suppression of AKT activation in bone marrow. (A) Western blots probing for p‐Akt and total Akt in bone marrow cells 1‐h after mice were injected via tail vein with Gedatolisib vehicle (0.3% lactic acid, 5% dextrose), 1 mg·kg−1 Gedatolisib or 10 mg·kg−1 Gedatolisib. (B) Analysis of band intensities (pAKT normalized by total AKT) presented for each treatment condition. *P = 0.012 when compared to vehicle by ANOVA and Dunnett’s post‐test. n = 3 femurs from three individual mice analyzed per treatment condition. Error bars represent SEM. (C) Western blots probing for p‐Akt and Total Akt in bone marrow cells two‐hours after mice were injected via tail vein with vehicle, 1 mg·kg−1 Gedatolisib or 10 mg·kg−1 Gedatolisib. (D) Analysis of band intensities (pAKT normalized by AKT) presented for each treatment condition. *P = 0.022 when compared to vehicle by ANOVA and Dunnett’s post‐test. Femurs from n = 3 mice analyzed per treatment condition. Error bars represent SEM. [file MOL2-16-130-s001.pdf]
